# Acute Effects of Percussive Massage Therapy on Thoracolumbar Fascia Thickness and Ultrasound Echo Intensity in Healthy Male Individuals: A Randomized Controlled Trial

**DOI:** 10.3390/ijerph20021073

**Published:** 2023-01-07

**Authors:** Chao Yang, Xingyu Huang, Ying Li, Wiraphong Sucharit, Patpiya Sirasaporn, Wichai Eungpinichpong

**Affiliations:** 1 Department of Exercise and Sport Sciences, Faculty of Graduate School, Khon Kaen University, Khon Kaen 40002, Thailand; yangchao@kkumail.com; 2 Research and Training Center for Enhancing Quality of Life of Working-Age People, Khon Kaen 40002, Thailand; 3 Department of Human Movement Sciences, Faculty of Physical Education, Gan Nan Normal University, Ganzhou 341000, China; gnsyhxy@sina.com; 4 School of Rehabilitation Medicine, Gan Nan Medical University, Ganzhou 341000, China; liyingganyi@163.com; 5 School of Physical Therapy, Faculty of Associated Medical Sciences, Khon Kaen University, Khon Kaen 40002, Thailand; wiraphongsu@kkumail.com; 6 Research Center in Back, Neck, Other Joint Pain and Human Performance (BNOJPH), Division of Physical Therapy, Faculty of Associated Medical Sciences, Khon Kaen University, Khon Kaen 40002, Thailand; 7 Department of Rehabilitation Medicine Faculty of Medicine, Khon Kaen University, Khon Kaen 40002, Thailand; spatpiya@kku.ac.th

**Keywords:** ultrasound, massage, percussion, fascia, back

## Abstract

Percussive massage therapy (PT) has been widely used by therapists and the fitness population to treat myofascial-related conditions. However, there is no evidence to confirm the effects of PT on the fascia. This study aimed to investigate the effects of PT on thoracolumbar fascia (TLF) morphology and other related outcomes. Methods: Sixty-six healthy males participated and were randomly allocated into a percussive massage group (PT group) and a control group. The PT group received 15 min of back percussion massage, while the control group rested prone lying in the same environment for 15 min. Thoracolumbar fascia (TLF) thickness and echo intensity, perceived stiffness, lumbar flexibility, and skin temperature were measured in both groups before and immediately after the intervention. Result: TLF thickness and lumbar flexibility did not change when compared in the two groups. However, the echo intensity (left side, difference −3.36, 95% CI −5.1 to −1.6; right side, difference −4.39, 95% CI −6.1 to −2.7) and perceived stiffness (difference, −1.18, 95% CI −1.84 to −0.52) in the TLF region were significantly lower in the PT group than in the control group and were accompanied by increased skin temperature (difference 0.29, 95% CI 0.11 to 0.48). Conclusion: We suggest that a 15 min PT with 30 Hz on the back region could reduce TLF echo intensity and perceived stiffness and increase skin temperature in healthy men individual.

## 1. Introduction

Fascia plays a potential role in developing chronic diseases [1], even cancer [2]. As the largest fascial structure in the body, the thoracolumbar fascia (TLF) is a composite structural material consisting of tendons and fascial planes that connects the latissimus dorsi and gluteus maximus muscles [3]. Its primary function is to help stabilize the spine and transmit forces between the lumbar spine and pelvis [4]. At the beginning of the twenty-first century, M. Langevin proposed an interactive model of the pathogenesis of thoracolumbar fascia and lower back pain [5]. A subsequent series of studies found that the thoracolumbar fascia in patients with low back pain showed thickening [6], reduced shear strain [7], and structural disturbances [8] compared to individuals without low back pain. These changes in the TLF may induce pain by stimulating A- and C-fiber nociceptors [9,10]. Fascial thickening has been found to vary, even in healthy individuals [11]. This is associated with fascial densification caused by overuse [12]. The abnormality of the fascial structure could be reversed by applying mechanical forces (twisting, tension, compression, stretching, bending, friction, and percussion) to the soft tissues [5,13,14]. This phenomenon may be attributed to mechanical stimulation that increases the local strain on the loose connective tissue, causing hyaluronic acid hydration to reduce the viscosity [15]. Treatment of deep fascia requires adequate and sustained pressure [16], thereby therapists often use power tools, such as handheld percussion devices, to improve fascial function [17,18].

Recently, handheld percussive massage therapy (PT) has received increasing attention. Different manufacturers (e.g., Theragun, Hyperice) offer this equipment for self-massage or therapist treatment, which can usually provide up to 53 Hz percussion frequency, 16 mm amplitude, and various massage heads that can be matched to different body parts. Due to its portability and stability, PT is favored by the physical therapist community and sports people [17]. Available evidence suggests that PT can increase range of motion (ROM), improve recovery, and reduce muscle pain and stiffness [19,20,21,22]. The mechanism may be related to continuous percussion stretching the muscle fiber, causing collagen remodeling and elastin changes [13,23], affecting the fascia’s densification, adhesions, and viscoelastic properties [12]. A review showed that most therapists use PT to treat myofascial syndrome [17]. Although percussion massage has been widely used in therapeutic practice, there are no relevant studies on whether PT can affect the fascial structure.

This study aims to investigate the effects of PT on TLF morphology and other related outcomes. We hypothesized that 15 min of PT would reduce participants’ TLF thickness, echo intensity of the ROI (range of interest) on TLF, and perceived stiffness, as well as increase skin temperature and lumbar flexibility.

## 2. Materials and Methods

### 2.1. Experimental Design

A randomized controlled trial was used to assess the immediate effects of PT on primary outcomes (fascia echostructure data) and secondary outcomes (perceived stiffness, skin temperature, lumbar flexibility). Data were collected before treatment (baseline) and immediately after the PT intervention. The study was registered with the Thailand Clinical Trials Registry (TCTR20221223001).

### 2.2. Sample Size Calculation

This study was a two-group parallel RCT design, and the main outcome was the change in TLF thickness before and after treatment. Paired *t*-test module of PASS software was used, according to alpha = 0.05 and power = 80%; the mean value of change in a TLF thickness before and after treatment was expected to be 0.10, the standard deviation was 0.2, and the ratio between the two groups was 1:1 to find that at least 27 cases needed to be included in each group, and the dropout rate was set at 10%, so a total of 66 participants were needed.

### 2.3. Participants

Participants were recruited from Gannan Medical University and the surrounding communities. Sixty-six male participants were recruited to participate in this study. Inclusion criteria included men aged 18–30 with a BMI < 28 kg/m^2^. Participants who had one of the following conditions were excluded: skeletal muscle disease, spine statics disorders (such as scoliosis, hyperlordosis), chronic disease, a history of back surgery, and received massage therapy within a week, and weight training on the back area within the last three months. Participants signed the informed consent document before the experiment. The study protocol was approved by the Ethics Committee of Khon Kaen University, Thailand (no. HE642185) and by the Ethics Committee of Gannan Medical University, China (no. 2022003). This study was conducted following the guidelines of the Declaration of Helsinki. An independent researcher numbered the recruited participants sequentially and placed them in opaque sealed envelopes; the randomization scheme was generated by using the website Randomization.com <http://www.randomization.com (accessed on 17 November 2022)>. Random block sizes were used to randomize participants into two groups: the PT group, *n* = 33, and the control group, *n* = 33. Participants could not be blinded based on their need to know the specific intervention method.

### 2.4. Intervention Protocol

**Figure 1:**
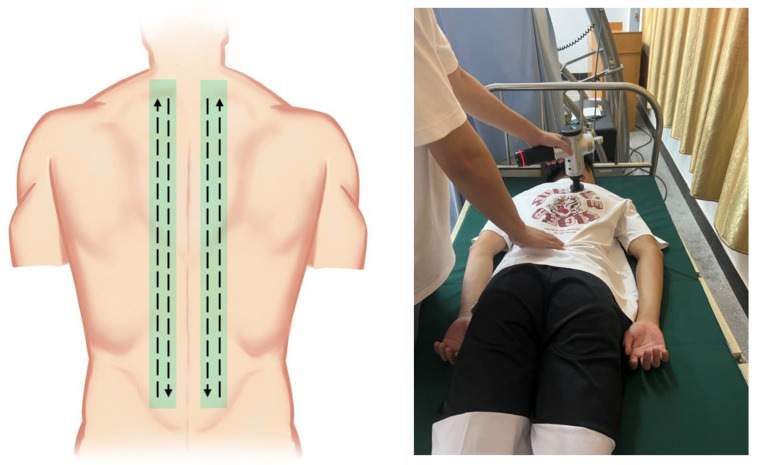
Percussive massage therapy diagram.

Participants in the PT group wore comfortable, close-fitting clothing and lay prone on a massage bed with their hands naturally resting on the side of their trunk. The PT protocol was based on our pilot study parameters for back percussion massage [24]. PT was performed by the same researcher using a hand-held massage gun (Hyperice, Irvine, CA, USA), tapped at a frequency of 30 Hz (rotational speed 1800 rpm/m) with a flat massage head. The duration of the percussive massage was 15 min. The use of this percussion frequency was based on the fact that a PT of 30 Hz is appropriate for myofascial release [25], and also that previous studies have found a 15 min duration of back massage to be beneficial [26,27]. The researcher placed the massage head vertically on the participants’ backs. They massaged the erector spinae muscle, moving from the forces on the lateral side of the erector spinae muscle in a straight line from the caudate to the cephalad. When it reached the vicinity of the scapula, the massage head was moved medially and in a straight line from distal to proximal along the longissimus dorsi and the musculus spinalis dorsi. This process was controlled at 25 s, following the percussion route (Figure 1), repeatedly for 7.5 min on each side of the back. The order of the left and the right back tapping massage was randomized; the researchers kept the massage gun in contact with the skin throughout the intervention and tried to apply the same pressure.

The participants in the control group did not receive any massage. They lay prone in the same position and environment as the PT group for 15 min.

### 2.5. Outcomes Measurement

#### 2.5.1. Ultrasound Imaging Assessment

**Figure 2:**
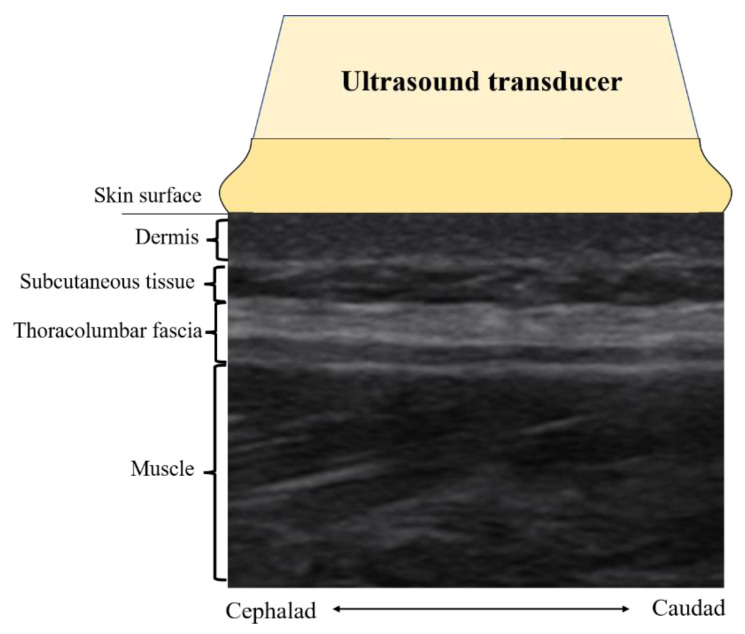
Example of a parasagittal ultrasound image.

All ultrasound images were acquired by a certified ultrasound clinician blinded for group allocation of the participants. A Mindray M7 scanner (Mindray, Shenzhen, China) with a 4 cm, 10 MHz linear array transducer was used for ultrasound B-mode imaging of the lumbar region. Bilateral parasagittal images were acquired with the probe centered at 2 cm lateral to the middle of the L2–3 interspinous ligament [6,7]. Figure 2 illustrates the ultrasound imaging acquired from this location. The TLF at this location is parallel to the skin. There is less angular variation between the skin surface and the TLF, which previous studies suggested to be a reliable method for measuring TLF thickness, and the intra-observer (ICC: 0.67–0.77) agreement and inter-observer (ICC: 0.82–0.92) agreement [28,29]. Finally, grayscale images were extracted and stored in Digital Imaging and Communications in Medicine (DICOM) format.

##### Fascial Echo Intensity Measures

**Figure 3:**
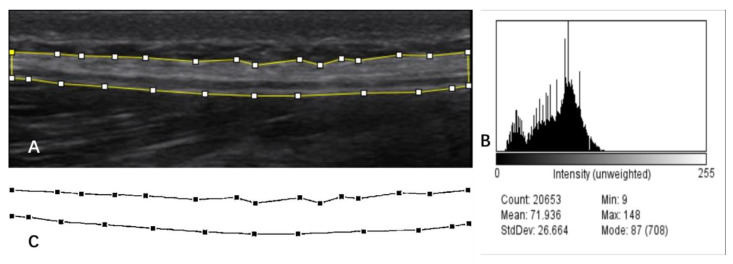
Thoracolumbar fascia thickness and echo intensity measurement process. (**A**). Use ImageJ to draw the boundaries of the TLF. (**B**). CSA of TLF histogram displayed. (**C**). Hyperbolic diagram of the TLF of CSA obtained after texture extraction by AI imaging software (version 1, Yongzhou, China).

TLF is a fibrous connective tissue composed of collagen (3) that appears as a highly echogenic layer in ultrasound images [30], and it has a clear border in contrast to subcutaneous tissue (fat) as well as muscle (Figure 2). As shown in Figure 3A, one researcher completed manual labeling of all TLF cross-sectional areas (CSA) using ImageJ software (United States National Institutes of Health; Bethesda, MD, USA). Offline assessment of the echogenicity and tissue homogeneity within TLF CSA (Figure 3B) were obtained, and the mean value of echo intensity of TLF CSA was taken for data analysis. This measurement of echo intensity has been widely used in skeletal muscle research [31].

##### Fascia Thickness Measures

Previous studies have mainly used the fascial thickness of a single location or the mean value of several areas for data analysis [8,29]. This does not give an actual average of TLF thickness. To obtain the average thickness of the TLF more accurately, we developed a Python-based method for collecting fascia thickness. First, get a hyperbolic diagram (Figure 3C) of the TLF of CSA obtained after texture extraction by AI imaging software (version 1. Yongzhou, China). Next, the image was converted to grayscale and binarized using Python programming (version 3.6.4). Programming with programming languages, each pixel on the grayscale image was sampled, and the distance between the top and bottom of the hyperbola was calculated and divided by the number of horizontal pixels (sampling points). The calculation result is based on the reference scale of the original image.

#### 2.5.2. Perceived Stiffness

All secondary outcomes were measured by two trained researchers who were informed of the subgroups. The perceived stiffness of the back was measured using a 10 cm visual analog scale. The left side anchored “no stiffness at all,” and the right side anchored “the strongest stiffness imaginable.” Participants were asked to draw a line across the VAS based on their self-perceived back condition [32].

#### 2.5.3. Skin Temperature

Skin temperature was measured by an infrared body surface thermometer (XIAOMI thermometer, Beijing, China). The measurement points were positioned in line with the ultrasound image scanning position. The researcher held the instrument so that the infrared measuring head was parallel to the measurement area and 3 cm apart and repeated the measurements three times to take the average value for data analysis. According to the product parameters of the instrument, the clinical repeatability was ±0.3 °C, and the resolution was 0.1 °C, which has good reliability.

#### 2.5.4. Lumbar Flexibility

The Schober test was used in this study to assess lumbar flexibility. The Schober test is a commonly used clinical measure for determining lumbar flexibility [33]. The modified Schober test demonstrated moderate validity (r = 0.67; 95% CI 0.44–0.84) when compared with X-ray, and excellent reliability (intra: ICC = 0.95; 95% CI 0.89–0.97; inter: ICC = 0.91; 95% CI 0.83–0.96) [34]. Participants’ feet were spaced approximately 30 cm apart, and a researcher first made a horizontal mark on subject L5 (fifth lumbar vertebra). Next, two horizontal marks were made 5 cm below and 10 cm above this point (a total distance of 15 cm). The patient was then asked to touch their toes while keeping the knee straight, and the distance between the upper and lower points was recorded with a soft ruler while the participant’s lumbar region was flexed. Measurements were repeated three times for averaging, with 30 s intervals between each measurement, and finally, the mean value was subtracted from the initial length (15 cm) for data analysis.

### 2.6. Statistics Analysis

Descriptive statistics were used for baseline demographic data and the results. Kolmogorov–Smirnov calculations were used to ensure a normal distribution of the data. Paired-sample *t*-tests were used to determine within-group differences between the pre-test and post-test for all parameters in the PT and control groups. One-way analysis of covariance was used to determine the differences in all parameters between the two groups after adjusting for pre-intervention baseline values. Residuals of the within/between-group dependent variables were found to be equivariant by plotting to scatter plots and performing Levene’s tests. In addition, there were no standardized residuals greater than 3 for the data in this study, suggesting no significant outliers. Differences were considered at the level of *p* < 0.05. Cohen’s d statistic was used to compute an effect size. The effect size less than 0.2 was considered to reflect a negligible difference, at least 0.2 to at most 0.5 a small difference, at least 0.5 to at most 0.8 a moderate difference, and at least 0.8 a large difference [35]. All calculations were performed using SPSS version 26.0 (SPSS Inc., Chicago, IL, USA).

## 3. Results

**Figure 4:**
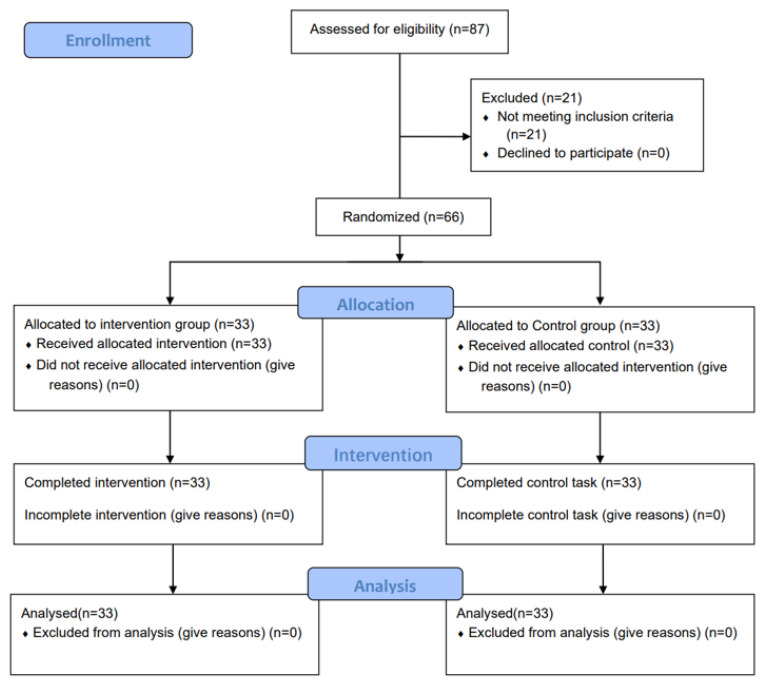
The CONSORT flow chart shows the acute percussive massage trial participant pathway. Intervention group = PT group.

Figure 4 presents a diagram of subject enrollment as per the guidelines of the Consolidated Standard of Reporting Trials (CONSORT).

All 66 participants completed the experiment with a mean age of 22 ± 4.1 years old, a height of 175.5 ± 5.7 cm, a weight of 69.7 ± 6.9 kg, and a BMI of 22.6 ± 1.8 kg/m^2^. No differences were found between the two groups regarding demographics (Table 1).

### 3.1. Echostructure Data

Inconsistent with our hypothesis, we did not observe any significant differences in fascial thickness between the two groups in intra-group (Table 2) and inter-group (Table 3) pre–post comparisons. The paired-sample *t*-test showed a substantial reduction in TLF CSA^EI^ (*p* < 0.001) in the percussive massage therapy group bilaterally compared to baseline values after percussion massage. In the control group, only a significant reduction in the right TLF (*p* < 0.05) was detected compared to before the intervention. ANCOVA analysis showed a subject effect for echo intensity of TLF CSA in both groups after the intervention (F = 14.7, *p* < 0.001), and post hoc analysis showed a significant reduction in echo intensity of TLF CSA bilaterally in the PT group compared to the control group (Table 3).

### 3.2. Secondary Outcomes

**Figure 5:**
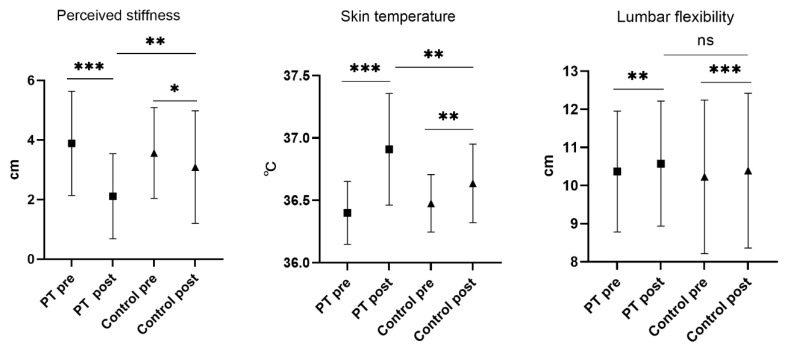
Results of inter-group and intra-group comparisons of other outcomes. Abbreviations: PT: percussive massage therapy; Control: control group; Pre: before the intervention; Post: after the intervention. * represents *p* < 0.05, ** represents *p* < 0.01, *** represents *p* < 0.001, ns represents no significant differences.

The paired-sample *t*-test showed that the perceived stiffness of the PT group decreased from 3.9 ± 1.8 cm to 2.1 ± 1.4 cm after the intervention (*p* < 0.001). Perceived stiffness also significantly reduced in the control group, from 3.6 ± 1.5 cm to 3.1 ± 1.9 cm (*p* < 0.01). In addition, both groups had significant differences in skin temperature and lumbar flexibility compared to the pre-intervention period (Figure 5). The ANOVA demonstrates the PT group had significantly lower perceived stiffness (*p* < 0.01) and substantially higher skin temperature (*p* < 0.01) after the intervention. No difference in lumbar flexibility was found between the two groups after the intervention (Figure 5).

## 4. Discussion

Essential to our knowledge, this is the first study to use ultrasound imaging to measure fascial structures as an indicator of the outcome of a percussion massage treatment intervention. The current study found that a 15 min PT did not change TLF thickness and lumbar flexibility. However, it increased the skin temperature of participants, as well as decreased the TLF echointensity and perceived stiffness.

The working mechanism of PT combines elements of percussive massage and vibration. On the one hand, PT can induce tonic vibration reflexes by vibrating at specific frequencies to reduce stress on muscles and connective tissues [36]. PT may also stretch muscle fibers through muscle waves generated by high-frequency percussion [23]. Therefore, PT has the potential to mechanically flatten and remove lipid tissue, release fascial restrictions, and change the mechanical properties of the fascia, such as density, stiffness, and viscosity [37]. Contrary to our hypothesis, TLF thickness did not change after PT. This is not unexpected: although, in previous studies, the reductions in post-treatment fascial thickness found were found in specific groups (e.g., chronic pain, fascial injury) [15,38], our participants were healthy adults who did not have high values of fascial thickness. The mean TLF thickness in the PT and control groups before the intervention was 2.55 mm and 2.63 mm, much lower than the TLF thickness recorded by M. Langevin with ultrasound imaging in men without lower back pain (*n* = 24), 37 ± 4 mm [7]. Therefore, the ceiling effect could influence the results of the current study. The lower TLF thickness observed in this study could be because our participants were, on average, 22.5 years younger than that in the previous research. In another study comparing fascial thickness differences between young and older adults, TLF was significantly thicker in older people than in younger people [29]. Aging may affect the extracellular interstitium of the myofascial, increase in thickness, and collagen interaction [39]. Moreover, perhaps we applied only one session of PT and did not find these acute effects.

Echo intensity is a first-order statistical quantitative ultrasound descriptor commonly used to assess tissue texture characteristics and quality by tissue echo [31]. In ultrasound images, fascial tissue (fibers) is rendered white (hyperechoic), and loose connective tissue contains high amounts of hyaluronic acid (HA). In a healthy state, HA has a low viscosity and appears black (hypoechoic) [40,41]. The high echo intensity value indicates high fibrous organization within the tissue, and HA becomes more viscous, like non-Newtonian fluids [12,42]. A significant finding was that the PT had reduced echo intensity of TLF CSA, although we did not find a change in fascial thickness. This might be related to the texture change in the loose connective tissue; stated differently, PT probably reduces the viscosity of HA. Several previous studies have evaluated the effects of myofascial release techniques and manipulative therapy on fascial shear and mobility and also have shown an association with hyaluronic acid in the loose connective tissue layer [15,43,44,45]. HA is a glycosaminoglycan (GAG) type, consisting of a linear non-sulfated polymer. In the immobile state the concentration of HA increases, which reduces the lubrication and gliding of the connective tissue and muscle layers [42,46]. Although the physiological mechanism behind this has not been clarified, it was found that the vertical vibration and pressure generated by PT squeezed HA flow toward the fascial rim region [47]. This mechanical stimulation led to greater lubrication and increased sliding between the fascial layers [48]. The other reason for the decrease in echo intensity after PT is probably due to the HA returning to its normal physiological state as the temperature increases. From a molecular point of view, the rise in HA viscosity is due to the entanglement of HA chains. However, when the temperature is increased and alkalinized, the HA chain is gradually depolymerized. This shows that the increase in temperature facilitates the reduction of HA viscosity [49]. In the present study, the increase in skin temperature after PT (*p* < 0.01, difference 0.29, 95% CI, 0.11–0.48) supported these early findings.

The results of ANCOVA demonstrated significantly lower perceived stiffness in the PT group after the intervention compared to the control group. A consideration is the stimulation of Ruffini cylinders and Pacinian vesicles in the fascia during PT, which may lead to inhibition of sympathetic activation and muscle relaxation [50]. Robert Schleip discovers that fascia has active contractility and can affect skeletal dynamics [51,52], which allows the morphology of fascia to directly affect flexibility [7,29]. Usually, improvements in muscle compliance affect flexibility; however, in the present study, no significant differences were found in lumbar flexibility between the PT and control groups. Andreas investigated the effects of PT on plantar flexor muscle range of motion (ROM) in 16 healthy male volunteers. He found that the maximum dorsiflexion ROM increased significantly by 18.4% after massage treatment [20]. Another study showed that 11 min local vibration with 20 Hz can improve back flexibility in older adults, young athletes, and college students [32]. There are two factors to consider: one is that the 30 Hz PT we used was much lower than the 53 Hz used in the Andreas study, and the second is that we only massaged the back, whereas, in the Siegmund investigation, the legs and hips, and back were intervened.

## 5. Limitation

This study has limitations, such as only using a percussion frequency of 30 Hz. It will be interesting to measure the effect of different frequencies and different location of PT on TLF in the future. Moreover, the secondary outcomes were performed by two researchers who were not blinded, and this study did not measure the physical activity level of participants, which carries a degree of risk of bias. Furthermore, the participants in this study were young, healthy males, so the results need to be generalized with caution for women and other populations. In future studies, validation of the long-term effects of PT on TLF in patients with chronic lower back pain is necessary because TLF in patients with LBP is fibrotic or dense due to inflammation, fatty infiltration, and adhesions.

## 6. Conclusions

This is the first study to find that PT possibly can affect fascial structures, which may provide some reference for PT users (therapists, general population). The results show that 15 min of 30 Hz PT did not reduce the TLF thickness in healthy adult males but decreased the echo intensity in the TLF region, probably due to a reduction in the viscosity of hyaluronic acid between the loose connective tissue. PT performed on the back alone did not facilitate lumbar flexibility but increased skin temperature and improved perceived stiffness. Therefore, it is recommended that PT be used to improve and prevent TLF densification in the healthy population.

## Figures and Tables

**Table 1:** Participants’ characteristics.

Characteristics	PT Group (*n* = 33)	Control Group (*n* = 33)
Age (years)	21.6 ± 4.4	22.6 ± 3.7
Height (cm)	176.2 ± 5.8	174.9 ± 5.6
Weight (kg)	68.9 ± 7.9	70.5 ± 5.9
BMI (kg/m^2^)	22.2 ± 1.9	23 ± 1.5

**Table 2:** Post differences of ultrasound data within groups using paired-sample *t*-test.

Outcomes	Group	Before	After	*p*-Value
Fascia thickness L (mm)	PT	2.56 ± 0.64	2.57 ± 0.63	0.835
	Control	2.56 ± 0.69	2.56 ± 0.66	1.000
Fascia thickness R (mm)	PT	2.54 ± 0.6	2.47 ± 0.59	0.127
	Control	2.69 ±0.67	2.67 ± 0.69	0.152
Echo intensity L (CSA^EI^)	PT	71.9 ± 9.7	68.5 ± 9.5	<0.001
	Control	73.9 ± 8	73.6 ± 8.1	0.270
Echo intensity R (CSA^EI^)	PT	73.7 ± 8.1	68.4 ± 9.2	<0.001
	Control	74.2 ± 8	73.4 ± 7.8	0.02

Note: Fascia thickness L: left side of thoracolumbar fascia thickness; Fascia thickness R: right side of thoracolumbar fascia thickness; CSA^EI^: echo intensity of cross-sectional areas. Data is presented as Mean ± SD (Standard deviation).

**Table 3:** Post differences of all the outcomes between groups, baseline adjusted using ANCOVA.

Outcomes	Group	Baseline		After Intervention		After Intervention (Adjusted)		Difference (95% CI)	*p*-Value	Effect Size
		Mean	SE	Mean	SE	Mean	SE			
Fascia thickness L (mm)	PT	2.56	0.111	2.57	0.109	2.57	0.031	0.009 (−0.079 to 0.097)	0.837	0.03
	CG	2.56	0.119	2.56	0.115	2.56	0.031			
Fascia thickness R (mm)	PT	2.54	0.105	2.47	0.102	2.55	0.035	−0.047 (−0.15 to 0.05)	0.349	0.12
	CG	2.69	0.117	2.67	0.119	2.59	0.035			
Echo intensity L (CSA^EI^)	PT	71.9	1.69	68.5	1.65	69.4	0.62	−3.36 (−5.1 to −1.6)	<0.001	0.48
	CG	73.9	1.39	73.9	1.39	72.7	0.62			
Echo intensity R (CSA^EI^)	PT	73.7	1.4	68.4	1.61	68.7	0.6	−4.39 (−6.1 to −2.7)	<0.001	0.64
	CG	74.2	1.39	73.4	1.36	73.1	0.6			
Perceived stiffness (cm)	PT	3.89	0.3	2.11	0.25	2.01	0.23	−1.18 (−1.84 to −0.52)	<0.001	0.45
	CG	3.56	0.27	3.09	0.33	3.19	0.23			
Skin temperature (°C)	PT	36.4	0.04	36.9	0.08	36.9	0.67	0.29 (0.11 to 0.48)	0.002	0.40
	CG	36.5	0.04	36.6	0.05	36.6	0.67			
Lumbar flexibility (cm)	PT	10.4	0.28	10.6	0.29	10.5	0.53	0.04 (−0.1 to 1.94)	0.550	0.08
	CG	10.2	0.35	10.4	0.35	10.4	0.53			

Note: Fascia thickness L: left side of thoracolumbar fascia thickness; Fascia thickness R: right side of thoracolumbar fascia thickness; CSA^EI^: echo intensity of cross-sectional areas; Data is presented as Mean ± SE: standard error mean; Difference (95% CI): 95% confidence interval for differences.

## Data Availability

Not applicable.
