# Correction: Yang et al. Acute Effects of Percussive Massage Therapy on Thoracolumbar Fascia Thickness and Ultrasound Echo Intensity in Healthy Male Individuals: A Randomized Controlled Trial. *Int. J. Environ. Res. Public Health* 2023, *20*, 1073

**DOI:** 10.3390/ijerph21121585

**Published:** 2024-11-28

**Authors:** Chao Yang, Xingyu Huang, Ying Li, Wiraphong Sucharit, Patpiya Sirasaporn, Wichai Eungpinichpong

**Affiliations:** 1 Department of Exercise and Sport Sciences, Faculty of Graduate School, Khon Kaen University, Khon Kaen 40002, Thailand; yangchao@kkumail.com; 2 Research and Training Center for Enhancing Quality of Life of Working-Age People, Khon Kaen 40002, Thailand; 3 Department of Human Movement Sciences, Faculty of Physical Education, Gan Nan Normal University, Ganzhou 341000, China; 4 School of Rehabilitation Medicine, Gan Nan Medical University, Ganzhou 341000, China; 5 School of Physical Therapy, Faculty of Associated Medical Sciences, Khon Kaen University, Khon Kaen 40002, Thailand; 6 Research Center in Back, Neck, Other Joint Pain and Human Performance (BNOJPH), Division of Physical Therapy, Faculty of Associated Medical Sciences, Khon Kaen University, Khon Kaen 40002, Thailand; 7 Department of Rehabilitation Medicine Faculty of Medicine, Khon Kaen University, Khon Kaen 40002, Thailand

There was an error in the original publication [1]. It was due to a decimal point error in converting the raw thoracolumbar fascia thickness data from centimeters to millimeters. The decimal point for thoracolumbar fascia thickness in Tables 2 and 3 was incorrect. The corrected Table 2 and Table 3 are shown below. And the sentence in Section 4. Discussion was corrected as follows: “The mean TLF thickness in the PT and control groups before the intervention was 2.55 mm and 2.63 mm”.

**Table 2:** Post differences of ultrasound data within groups using paired-sample *t*-test.

Outcomes	Group	Before	After	*p*-Value
Fascia thickness L (mm)	PT	2.56 ± 0.64	2.57 ± 0.63	0.835
	Control	2.56 ± 0.69	2.56 ± 0.66	1.000
Fascia thickness R (mm)	PT	2.54 ± 0.6	2.47 ± 0.59	0.127
	Control	2.69 ±0.67	2.67 ± 0.69	0.152
Echo intensity L (CSA^EI^)	PT	71.9 ± 9.7	68.5 ± 9.5	<0.001
	Control	73.9 ± 8	73.6 ± 8.1	0.270
Echo intensity R (CSA^EI^)	PT	73.7 ± 8.1	68.4 ± 9.2	<0.001
	Control	74.2 ± 8	73.4 ± 7.8	0.02

Note: Fascia thickness L: left side of thoracolumbar fascia thickness; Fascia thickness R: right side of thoracolumbar fascia thickness; CSA^EI^: echo intensity of cross-sectional areas. Data is presented as Mean ± SD (Standard deviation).

**Table 3:** Post differences of all the outcomes between groups, baseline adjusted using ANCOVA.

Outcomes	Group	Baseline		After Intervention		After Intervention (Adjusted)		Difference (95% CI)	*p*-Value	Effect Size
		Mean	SE	Mean	SE	Mean	SE			
Fascia thickness L (mm)	PT	2.56	0.111	2.57	0.109	2.57	0.031	0.009 (−0.079 to 0.097)	0.837	0.03
	CG	2.56	0.119	2.56	0.115	2.56	0.031			
Fascia thickness R (mm)	PT	2.54	0.105	2.47	0.102	2.55	0.035	−0.047 (−0.15 to 0.05)	0.349	0.12
	CG	2.69	0.117	2.67	0.119	2.59	0.035			
Echo intensity L (CSA^EI^)	PT	71.9	1.69	68.5	1.65	69.4	0.62	−3.36 (−5.1 to −1.6)	<0.001	0.48
	CG	73.9	1.39	73.9	1.39	72.7	0.62			
Echo intensity R (CSA^EI^)	PT	73.7	1.4	68.4	1.61	68.7	0.6	−4.39 (−6.1 to −2.7)	<0.001	0.64
	CG	74.2	1.39	73.4	1.36	73.1	0.6			
Perceived stiffness (cm)	PT	3.89	0.3	2.11	0.25	2.01	0.23	−1.18 (−1.84 to −0.52)	<0.001	0.45
	CG	3.56	0.27	3.09	0.33	3.19	0.23			
Skin temperature (°C)	PT	36.4	0.04	36.9	0.08	36.9	0.67	0.29 (0.11 to 0.48)	0.002	0.40
	CG	36.5	0.04	36.6	0.05	36.6	0.67			
Lumbar flexibility (cm)	PT	10.4	0.28	10.6	0.29	10.5	0.53	0.04 (−0.1 to 1.94)	0.550	0.08
	CG	10.2	0.35	10.4	0.35	10.4	0.53			

Note: Fascia thickness L: left side of thoracolumbar fascia thickness; Fascia thickness R: right side of thoracolumbar fascia thickness; CSA^EI^: echo intensity of cross-sectional areas; Data is presented as Mean ± SE: standard error mean; Difference (95% CI): 95% confidence interval for differences.

The authors apologize for the inconvenience; this correction did not affect the results or conclusions of this study. This correction was approved by the Academic Editor. The original publication has also been updated.
